# Pyrethroid resistance alters the blood-feeding behavior in Puerto Rican *Aedes aegypti* mosquitoes exposed to treated fabric

**DOI:** 10.1371/journal.pntd.0005954

**Published:** 2017-09-20

**Authors:** Natasha M. Agramonte, Jeffrey R. Bloomquist, Ulrich R. Bernier

**Affiliations:** 1 Emerging Pathogens Institute, Department of Entomology & Nematology, University of Florida, Gainesville, Florida, United States of America; 2 U.S. Department of Agriculture, Agricultural Research Service, Center for Medical, Agricultural, and Veterinary Entomology, Gainesville, Florida, United States of America; North Carolina State University, UNITED STATES

## Abstract

Emerging insecticide resistance is a major issue for vector control. It decreases the effectiveness of insecticides, thereby requiring greater quantities for comparable control with a net increase in risk of disease resurgence, product cost, and damage risk to the ecosystem. Pyrethroid resistance has been documented in Puerto Rican populations of *Aedes aegypti* (L.) mosquitoes. In this study, topical toxicity of five insecticides (permethrin, etofenprox, deltamethrin, DDT, transfluthrin) was determined for susceptible (Orlando—ORL) and resistant (Puerto Rico—PR) strains of *Ae*. *aegypti*. Resistance ratios were calculated using LD_50_ values, and high resistance ratios for permethrin (112) and etofenprox (228) were observed for the Puerto Rico strain. Behavioral differences in blood-feeding activity for pyrethroid-resistant and pyrethroid-susceptible strains of *Ae*. *aegypti* when exposed to pyrethroid-treated cloth were also explored. Strains were exposed for 15 min to a range of concentrations of pyrethroid-treated uniform fabric in a cage that contained 60 female *Ae*. *aegypti* mosquitoes. Interestingly, the resistance ratios for blood-feeding were similar for permethrin (61) and etofenprox (70), but were lower than their respective resistance ratios for topical toxicity, suggesting that knockdown resistance was the primary mechanism of resistance in the blood feeding assays. Results showed a rightward shift in the dose-response curves for blood-feeding that indicated higher concentrations of pyrethroids were necessary to deter blood-feeding behavior in the pyrethroid-resistant Puerto Rican strain of *Ae*. *aegypti*.

## Author summary

*Aedes aegypti* is a competent vector of mosquito-borne diseases and is the primary transmitter of yellow fever, zika, chikungunya, and dengue viruses. Through repeated insecticide treatments over the years, many populations of mosquitoes have developed resistance. Pyrethroid resistance is widespread in *Aedes aegypti* (L.) and has been problematic in Puerto Rico for decades. Using a pyrethroid-susceptible and a pyrethroid-resistant strain of *Ae*. *aegypti*, we created dose-response curves for feeding behavior using fabric treated with four distinct but related insecticides. Resistance ratios were calculated by dividing the LD_50_ values for the resistant strain by the susceptible LD_50_ values. We also calculated resistance ratios based on topical treatment, since the total amount of insecticides that the mosquitoes were exposed to in the topical assay could be controlled and compared to the blood-feeding assay. Interestingly, the resistance ratios for the blood-feeding were similar for permethrin and etofenprox, but lower than their respective resistance ratios for topical toxicity. Results from these assays showed a shift in the dose-response curves for blood-feeding amongst the susceptible and resistant strains, which indicated higher concentrations of pyrethroid chemicals necessary to deter blood-feeding behavior in the pyrethroid-resistant Puerto Rican strain of *Ae*. *aegypti*.

## Introduction

Insecticide resistance is a serious problem for vector control, and is associated with a higher cost and greater amounts of applied chemical to achieve a comparable level of control for a population of insects. Ultimately, resistance renders the insecticide being less effective for vector control, which unless other control methods are implemented, will increase both vector population size as well as disease transmission [1]. Levels of resistance lower than 10% will generally not affect disease control efforts [1]. It is important to identify and characterize developing resistance issues so that future control strategies can be optimized, or if there is evidence of resistance that adversely affects control, perhaps change or rotate the insecticide class [2,3].

Pyrethroids are acute neurotoxicants with low mammalian toxicity, low water solubility, and a high affinity to bind to sediment particles that are subdivided into two types, Type I and Type II, based on difference in structure and properties that relate to their intoxication [4,5,6]. Type I pyrethroids are more varied structurally with a wide variety of different alcohol groups. Etofenprox, which lacks the central ester group, is considered a Type I, non-ester pyrethroid [7] or a pseudopyrethroid [8]. Type II pyrethroids are characterized by the presence of an α-cyano group attached to the 3-phenoxybenzyl alcohol [9,4]. The inclusion of the α -cyano substituent produces greater insecticidal potency than Type I (permethrin), but with similar photostability [7].

Pyrethroids bind to and prevent the inactivation of sodium channels in neuronal membranes [7]. They are commonly used to control mosquito vector populations in areas of the world that suffer from mosquito-borne diseases, such as dengue and malaria [10,11]. Through repeated treatments over multiple years, some populations of mosquitoes have developed broad cross-resistance to other chemicals in this group, since they have a similar mode of action. A study done in 1989 documented resistance to pyrethroids in Puerto Rican *Aedes aegypti* (L.) mosquitoes, but found no evidence for resistance to organophosphates or carbamates, despite exposure to ground and aerial ULV applications of malathion [12].

Dengue is endemic in Puerto Rico, as is *Ae*. *aegypti*, the principal mosquito vector of dengue, zika, chikungunya, and yellow fever viruses, all of which cause severe human morbidity and mortality [13]. Dengue control in Puerto Rico began in 1963, when an epidemic of dengue-3 virus resulted in 27,000 reported cases [14,15]. Since 1963, Puerto Rico has experienced epidemic dengue activity periodically and it continues to be a serious problem [16]. In this study, differences in blood-feeding activity for pyrethroid-resistant and pyrethroid-susceptible strains of *Ae*. *aegypti* when exposed to pyrethroid-treated cloth were explored and compared with the topical toxicity for five insecticides.

## Materials and methods

### Mosquitoes

Adult mosquitoes used in all bioassays were female *Ae*. *aegypti*. The Orlando strain mosquitoes (ORL) were obtained from the colony maintained at the US Department of Agriculture, Agricultural Research Service, Center for Medical, Agricultural and Veterinary Entomology (USDA-ARS-CMAVE) laboratory in Gainesville, FL which originated in Orlando in 1952. The pyrethroid-resistant Puerto Rican strain (PR) of *Ae*. *aegypti* (NR-48830, BEI Resources, Atlanta, GA) was originally started from eggs collected in San Juan, Puerto Rico in June 2012 and this colony was also maintained at the USDA-ARS-CMAVE laboratory in Gainesville. This strain was challenged with permethrin as necessary to maintain the initial level of permethrin resistance (100 ppm exposure of permethrin to 3^rd^ larval instars of approximately every 3^rd^ generation) and the 5-8^th^ generations of mosquitoes were used for bioassays. Nulliparous mosquitoes aged 6–10 days were maintained *ad libitum* on a 10% sucrose solution at 25–28°C, 60–80% RH and a 14:10 (L:D) photoperiod. Nulliparous female mosquitoes aged 6–10 days were pre-selected for host seeking behavior from stock cages using a hand-draw box and a collection trap [17].

### Insecticides and dilutions

Anhydrous ethanol (Acros, CAS#64-17-5) or acetone (99.7%, Fisher Chemical, CAS#67-64-1) was used as the solvent for all chemicals, as well as the negative control. The insecticides used for assays were technical grade permethrin (95.3%, AMVAC Chemical, CAS#52645-53-1), etofenprox (97%, Landis Intl., Inc., CAS#80844-07-1), deltamethrin (99.6%, Fluka Analytical, CAS#52918-63-5), DDT (10.2% *o*,*p*’, 88.5% *p*,*p*’, ChemService, CAS#50-29-3), and transfluthrin (98%, Bayer, CAS#118712-89-3). Stock solutions of these insecticides were prepared, stored in a -8°C refrigerator, and at least five dilutions (10^2^−10^−3^ ng/mL) were made with ethanol within one week of testing.

### Topical toxicology insecticide assay

Mosquitoes were cold-anesthetized in a freezer at approximately -10°C, maintained anesthetized in a glass Petri dish on a portable chill table (Bioquip, Rancho Dominguez, CA) at about -4°C, separated into groups of 10 females, and each chemical dose was applied in triplicate (n = 30). Control group mosquitoes included both untreated mosquitoes as well as ethanol-treated mosquitoes (n = 60). Topical dosing of insecticides, from lowest concentration to highest (10^−5^–10^2^ ng/mg mosquito), was applied using a Hamilton repeating dispenser (PB600-1 1700 Series, Hamilton, Reno, NV) and 10 μL syringe over a piece of filter paper that was replaced following each insecticide application. A 200 nL droplet of each chemical dose was applied topically to the thorax of each mosquito. Dosed mosquitoes were placed into clear, labeled 103.5 mL cups, covered with a square piece of gauze fabric and secured with a rubber band. The syringe was rinsed with 30 volumes of acetone between each insecticide application. Dosed mosquitoes were held overnight at 25°C, 60% RH and provided with a 10% sugar solution-soaked cotton ball that was replaced daily. Mortality was assessed for each dose replicate at 24 h and 48 h to determine the number of dead or impaired mosquitoes, characterized by twitching and erratic or upside-down flight. Control mortality above 20% resulted in that replicate being retested. Replicates with control mortality below 20% were corrected using Abbott’s formula [18].

### Treated-uniform blood-feeding assay

A bolt of untreated Flame-Resistant Army Combat Uniform (FRACU Type III) fabric was provided by the US Army Natick Soldier Research, Development, and Engineering Center. The FRACU Type III cloth is currently the most common fabric construction used in US Army combat uniforms. Uniform fabrics were cut and sewn into sleeves with a surface area of approximately 690 cm^2^ for testing on the forearms of human volunteers. Six doses (10^1^−10^−5^ mg/cm^2^) of each pyrethroid insecticide treatment were diluted in 12 mL of acetone. Sewn uniform sleeves were rolled, placed into a sealed 250 mL amber jar, and vortexed vigorously to absorb the full amount of pre-measured insecticide. The uniforms were removed from the jar and air dried under a fume hood for 15 min to allow the acetone to evaporate.

**Fig 1:**
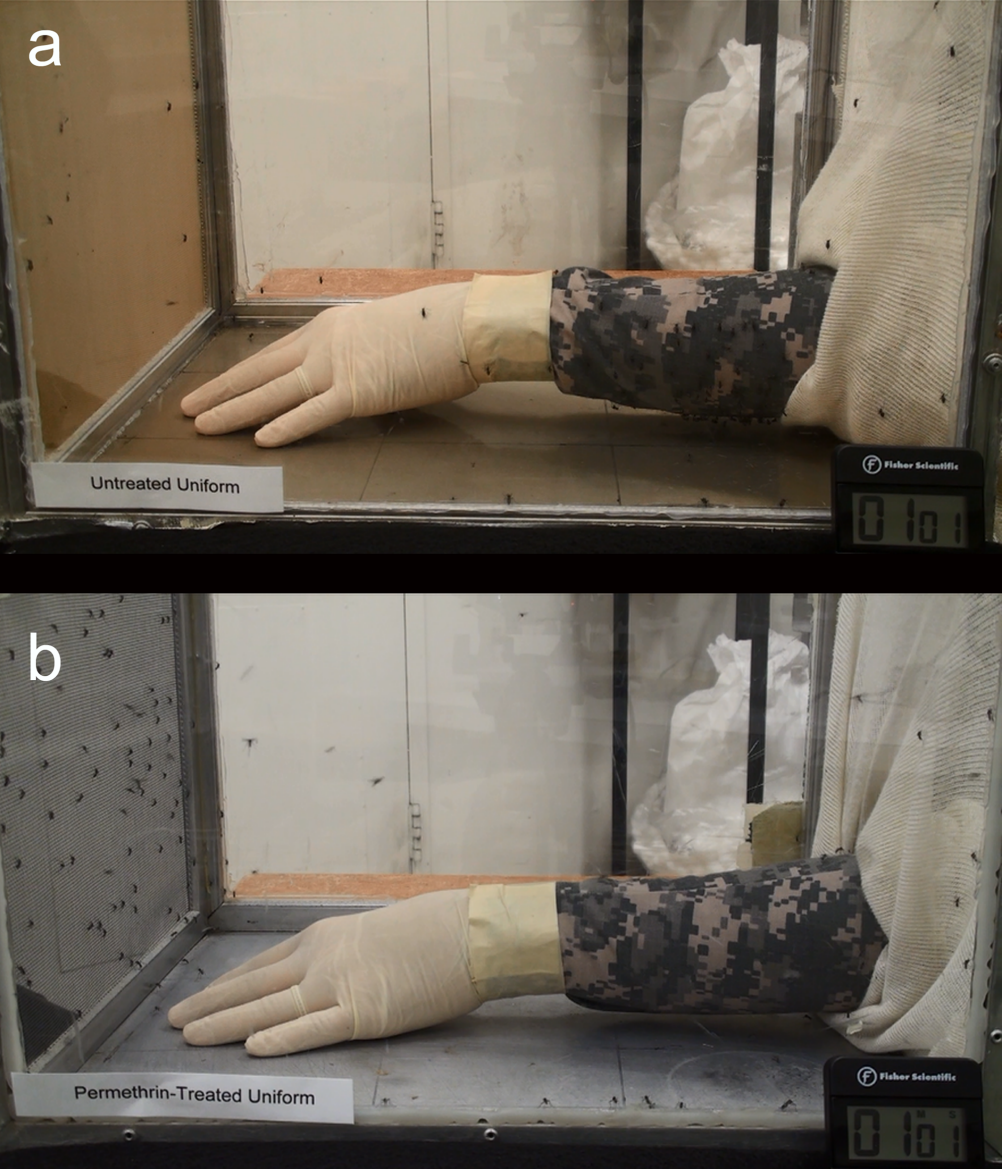
Example of blood-feeding assay with untreated control (A) and permethrin-treated uniforms (B) inserted into a stock cage filled with approximately 60 female *Ae*. *aegypti* mosquitoes. Photos by G. Allen.

For these assays, a volunteer’s hands were gloved and a dried sleeve was pulled tightly onto each arm, secured with masking tape at the wrist, and inserted into a stock cage filled with approximately 60 female *Ae*. *aegypti* mosquitoes for a 15 min duration test. An untreated control sleeve of the same fabric was paired with each treated uniform sleeve, in order to have a proper basis of comparison, as many uniform fabrics differ in weave tightness, which affects how easily a mosquito can penetrate the fabric (Fig 1). Blood-fed mosquitoes for each treatment concentration were recorded and compared to the total number of blood-fed mosquitoes for the fabric control. Assay results were averaged across 6 volunteers, with at least six treatment concentrations of insecticide per assay.

### Ethics statement

Volunteers for all bioassays were adults and provided a written, informed consent for enrollment in the study, approved by the University of Florida IRB-01 (Project # 69–2006).

### Statistical analysis

Percent bite protection (corrected for controls) was calculated using Abbott’s formula: [(C-T)/C]*100, where C = # mosquitoes blood-fed on control and T = # mosquitoes blood-fed on a treated sleeve. Data for mortality and bite protection were log transformed and normalized using the GraphPad Prism 6.02 software package [19]. Nonlinear regression analysis was performed in GraphPad Prism 6.02 software using a sigmoidal, four-parameter dose-response fit with a variable slope to compare the dose-response curves for each strain. The equation used for the nonlinear regression fit was: Y = Bottom + (Top-Bottom)/(1+10^((LogED50-X)*HillSlope)), where Y = Abbott corrected mosquito mortality, and X = Dose in ng/mg mosquito. The four parameters for the model fit include the Y maximum (Top), the Y minimum (Bottom), the log X value at 50 percent response (LogED50), and the slope (HillSlope). From these curve fits, ED_50_ estimates with 95% confidence limits, etc. were generated from Prism. The significance of the model term effects was evaluated using an F-test with a significance level of α = 0.05. Resistance ratios were calculated by dividing PR resistant strain LD_50_ (toxicity) or ED_50_ (blood feeding) values by the corresponding values generated for the susceptible ORL strain.

## Results

Comparisons of topical treatments between ORL and PR mosquito strains were made using LD_50_ estimates, calculated from the nonlinear regression fit of sigmoidal variable-slope dose-response curves for permethrin, etofenprox, deltamethrin, DDT, and transfluthrin (Table 1). The rank order of toxicity (Table 1) against ORL adult females by topical treatment was: deltamethrin >> permethrin > etofenprox > transfluthrin > DDT. Deltamethrin was 85-fold more active than permethrin, whereas the other differences in rank order of toxicity differed by only 2- to 3-fold (Table 1).

**Table 1:** Topical LD_50_, blood-feeding ED_50_, and resistance ratios (RR) for ORL and PR *Ae*. *aegypti* strains tested with five insecticides. Blood-feeding data for transfluthrin are omitted because it had slope factors not significantly different from zero.

Treatment (strain)	Topical LD_50_, ng/mg (95% CL)	Slope ± SEM	RR	Blood-feeding ED_50_, mg/cm^2^ (95% CL)	Slope ± SEM	RR
**Permethrin (ORL)**	0.017 (0.014–0.022)	1.005 ± 0.359		0.0007 (0.0004–0.0013)	0.526 ± 0.040	
Permethrin (PR)	1.9 (1.3–2.7)	0.907 ± 0.162	112	0.043 (0.011–0.161)	0.423 ± 0.076	61
**Etofenprox (ORL)**	0.032 (0.022–0.047)	1.157 ± 0.713		0.003 (0.002–0.007)	0.457 ± 0.047	
**Etofenprox (PR)**	7.3 (6.2–8.6)	0.911 ± 0.156	228	0.21 (0.04–1.08)	0.378 ± 0.136	70
**Deltamethrin (ORL)**	0.0002 (0.0001–0.0003)	1.336 ± 0.226		0.0002 (0.00008–0.0006)	0.350 ± 0.047	
**Deltamethrin (PR)**	0.13 (0.06–0.28)	0.481 ± 0.379	650	0.139 (0.052–0.371)	0.395 ± 0.054	695
**DDT (ORL)**	0.43 (0.31–0.59)	1.247 ± 0.318		0.072 (0.014–0.386)	0.187 ± 0.041	
**DDT (PR)**	6.7 (2.6–17.3)	0.562 ± 0.177	16	3.51 (0.12–103.1)	0.207 ± 0.085	49
**Transfluthrin (ORL)**	0.1 (0.07–0.13)	1.737 ± 0.291		NA		
**Transfluthrin (PR)**	2.9 (2.1–4.0)	0.809 ± 0.087	29	NA		

For PR, the rank order of toxicity changed dramatically and was: deltamethrin 15-fold > permethrin = transfluthrin > DDT = etofenprox (Table 1). F tests comparing the logLD_50_ values indicated that the ORL strain LD_50_s were significantly lower than the PR strain. The following statistical values were calculated for permethrin, where F (1,35) = 175.1, p < 0.0001 (α = 0.05); for etofenprox, F (1,35) = 321.7, p < 0.0001 (α = 0.05); for deltamethrin, F (1,35) = 74.6, p < 0.0001 (α = 0.05); for DDT, F (1,32) = 19.00 p = 0.0001 (α = 0.05); and for transfluthrin, F (1,32) = 110.09, p < 0.0001 (α = 0.05). The LD_50_ values for permethrin on ORL and PR resulted in a resistance ratio of 112, for etofenprox 228, for deltamethrin 650, for DDT 16, and for transfluthrin 29 (Table 1).

**Fig 2:**
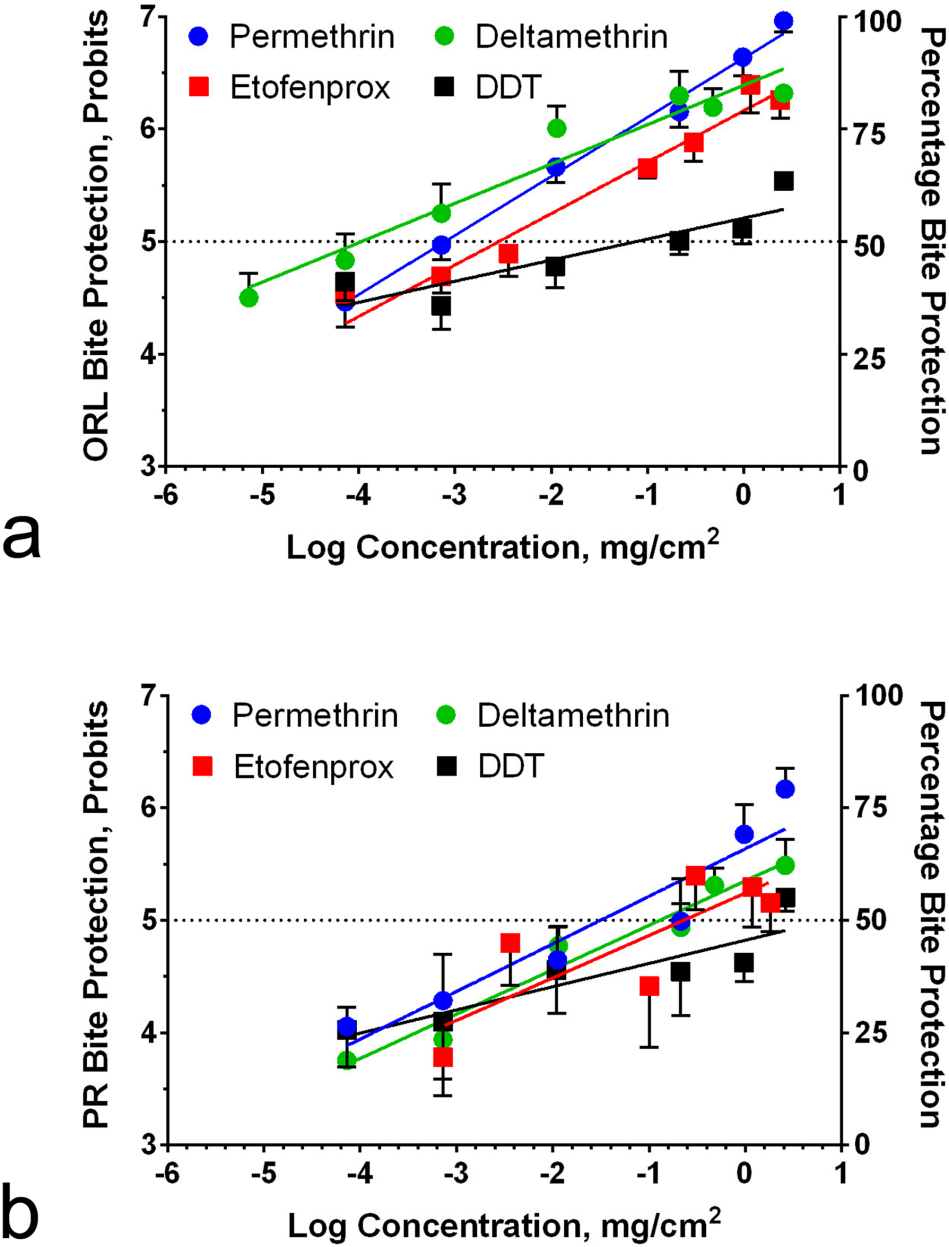
Dose response curves for blood-feeding behavior on permethrin-, etofenprox-, deltamethrin-, and DDT-treated uniforms with *Ae*. *aegypti* mosquitoes of the susceptible Orlando (ORL) strain (A) and pyrethroid-resistant Puerto Rico (PR) strain (B). The axes indicate the relationship between percent bite protection (right y-axis) and the log of the concentration in milligram per centimeter squared (x-axis) of insecticide-treated fabric. The left y-axis has been converted to a probit scale for clearer presentation. Error bars indicate SEM.

Comparisons in blood-feeding success following treatments with these same compounds to the ORL and PR strains were made using ED_50_ estimates, calculated from the linear regression fit of probit dose-response curves for permethrin, etofenprox, deltamethrin, and DDT (Table 1, Fig 2). The rank order of bite protection performance (Table 1) was much different from that observed for toxicity against PR adult females by treated sleeves: deltamethrin > permethrin > etofenprox > DDT. The difference between the pyrethroids was about 4-fold, while etofenprox was 24-fold more active than DDT (Table 1).

For PR, the rank order for bite protection was permethrin > deltamethrin > etofenprox > DDT (Table 1). F tests comparing the logED_50_ values indicated that the ORL strain ED_50_s were significantly lower than the PR strain. Statistical values for blood feeding protection in the two strains were calculated for permethrin, where F (1,66) = 29.6, p < 0.0001 (α = 0.05); for etofenprox, F (1,66) = 23.0, p < 0.0001 (α = 0.05); for deltamethrin, F (1,69) = 77.9, p < 0.0001 (α = 0.05); and for DDT, F (1,66) = 4.23, p = 0.044 (α = 0.05). The ED_50_ values for permethrin on ORL and PR resulted in a resistance ratio of 61, for etofenprox 70, for deltamethrin 695, and for DDT 49 (Table 1). Transfluthrin was omitted from these analyses because its response was variable in this assay, with slope values that did not differ significantly from zero.

## Discussion

Results from both the topical toxicity assays and the blood-feeding assays showed a rightward shift in the dose-response curves for the Puerto Rican strain of *Ae*. *aegypti*, which indicated higher concentrations of pyrethroid chemicals are necessary to deter the pyrethroid-resistant mosquitoes. This observation was true of all the compounds investigated, although the size of the shift varied by chemical treatment. As expected, there was significant resistance to permethrin in the PR strain, as permethrin resistance was previously documented in wild Puerto Rican mosquitoes aged 1 day with the standard WHO paper test [12]. Reid *et al*. [20] found a topical resistance ratio of 73 for permethrin for this strain of *Ae*. *aegypti* aged 2–5 days, and 33 of 164 cytochrome P450s tested were found to be significantly upregulated. When pre-treated with piperonyl butoxide, the permethrin resistance ratio was reduced to 15, suggesting that oxidative metabolic resistance accounted for about 5-fold of the resistance noted in the PR strain. Moreover, at least two *kdr* mutations were detected for this strain: Phe1534Cys and Val1016Iso [21]. These two mutations, associated with pyrethroid resistance in *Ae*. *aegypti*, are widespread in Latin America and the Caribbean [22]. More recent work with these strains by Estep et al. [21] found resistance ratios for permethrin (135) and DDT (19) which agree with the results found in this study for permethrin (112) and DDT (16). Resistance ratios for blood-feeding with permethrin and etofenprox are similar, but much lower than for their respective topical toxicities. The insecticides were applied directly to the mosquitoes for the topical toxicity assay and evaluated for lethality 24 hrs later, whereas, in the blood-feeding assay, knockdown and excitorepellent action were thought to play the dominant role in blood-feeding deterrence. Consequently, it is logical to conclude that the resistance ratios in the blood feeding assay would primarily reflect the presence of *kdr*, while the much greater lethality ratios in the topical toxicity assay would include the additional impact of metabolism and perhaps other mechanisms, such as penetration.

Etofenprox was overall a less effective insecticide than permethrin, as shown by the higher LD_50_ and ED_50_ results (Table 1). It also has a lower acute mammalian oral LD_50_ of >10,000 mg/kg compared to 500 mg/kg for permethrin [23]. Based on the acute toxicity of the active ingredients, the safety factor (oral LD_50_ for a 10-kg child /amount required for the treatment of a single bednet) is higher for etofenprox (133) compared to permethrin (0.7) [23]. As a fabric treatment, etofenprox may therefore have some advantages because of its lower mammalian toxicity, allowing for more chemical to be used safely. For surface contact exposures to the mosquito, an increase in the dosage rate would likely negate the reduced efficacy of etofenprox. However, since etofenprox had similar efficacy to permethrin in the blood-feeding assay, an increase in dosage as a fabric treatment may provide increased protection from blood-feeding for pyrethroid-resistant strains of mosquitoes.

Resistance ratios for blood-feeding with deltamethrin were comparable to its respective topical toxicities. Despite having the largest resistance ratio between the two strains in both assays, deltamethrin had the lowest amount of chemical needed for the LD_50_ topical treatment against the resistant strain of all the treatments examined. On a per gram basis, deltamethrin was the most cost effective treatment against both the susceptible ORL strain and the resistant PR strain of all the treatments examined, based on the ED_50_ quantities and an active ingredient cost comparison [24]. The resistance ratios of deltamethrin were similar in both assays. As a Type II pyrethroid, deltamethrin is known to have some excitorepellent properties in *Ae*. *aegypti* and Anopheline mosquitoes, but less so when contact irritancy is absent [25,26,27,28,29].

Resistance ratios for both blood-feeding and topical toxicity were low for DDT, and of about the same magnitude. However, the negative public perception and current banned status of DDT limits its usefulness as either a spray or fabric treatment for mosquito control in the US, although it is World Health Organization Pesticide Evaluation Scheme (WHOPES)-approved for malaria control [10,11]. Both the greater mosquito activity, and lower mammalian toxicity of pyrethroids makes them more attractive than DDT for widespread use, and ideally, insecticides of a different chemical class and with a different mode of action should be used in areas with ongoing pyrethroid resistance, if possible.

Although transfluthrin was initially examined for both topical toxicity and blood-feeding behavior, only the topical toxicity data are reported here. In the topical assay, transfluthrin was much less active than the other pyrethroids. This may be due to its rapid volatilization at ambient temperature, characteristic of a spatial repellent [30,31], which may have volatilized the small volumes of active ingredient used in topical treatment off of the cuticle before it could be absorbed. Another study by Wagman et al. [32] found that *Ae*. *aegypti* mosquitoes insensitive to pyrethroid repellents and containing the Val1016Iso *kdr* mutation also displayed decreased toxicity to transfluthrin and that this trait was heritable. Typically, there is a biological fitness cost to an organism associated with maintaining resistance mechanisms in the absence of an exposure [33]. This is especially true for multiple resistance mechanisms, as in this case with pyrethroid and carbamate insecticides, unless there is consistent exposure to both of these chemical classes. Evidence for an increase in multi-resistance has been noted as control programs make sequential use of one chemical class after another [1]. Interestingly, a study by Saavedra-Rodriguez *et al*. [34] with *Ae*. *aegypti* showed that the lineages with the highest frequencies of the *kdr* mutation resulted in a lower number of altered detoxifying genes. These results strongly suggest that this *kdr* mutation had a lower fitness cost compared to the metabolic resistance genes [33].

Specific applications of this work would apply most directly to military uniforms, which currently use only permethrin as a clothing treatment [35]. However, commercially available clothing for outdoor use is also limited only to permethrin [35], while insecticide-treated bednets are limited to permethrin, cypermethrin, and deltamethrin [36]. Future work should examine additional insecticides of different chemical classes and with different modes of action to be used in areas with ongoing pyrethroid resistance.

## Supporting information

S1 Table Abbott corrected percent mortaility by treatment type and mosquito strain for all replicates combined in the topical toxicity bioassays. (PDF)

S2 Table Percent bite protection by treatment type and mosquito strain for all replicates combined in the blood-feeding bioassays. (PDF)
