# Supplementary material for: Pyrethroid resistance alters the blood-feeding behavior in Puerto Rican Aedes aegypti mosquitoes exposed to treated fabric
Source: PLoS Negl Trop Dis. 2017 Sep 20;11(9):e0005954. doi: 10.1371/journal.pntd.0005954 (PMC5624645; doi:10.1371/journal.pntd.0005954)
Supplement: S1 Table — (PDF) [file pntd.0005954.s001.pdf]

S1 Table. Abbott corrected percent mortality by treatment type and mosquito strain for all replicates combined in the topical toxicity bioassays.

Topical Treatment: Permethrin
Strain: ORL

Treatment Active Ingredient (ng)	Log Treatment	Treatment (ng/mg mosquito)	Alive	Total	% Mortality - Abbott Corrected
0.000		0.000	9.167	10.000	**0.000**
0.004	-2.653	0.002	8.333	10.000	**9.091**
0.020	-1.954	0.011	6.333	10.000	**30.909**
0.040	-1.653	0.022	4.000	10.000	**56.364**
0.200	-0.954	0.111	0.000	10.000	**100.000**
0.400	-0.653	0.222	0.000	10.000	**100.000**
4.000	0.347	2.222	0.000	10.000	**100.000**

Topical Treatment: Permethrin
Strain: PR F7

Treatment Active Ingredient (ng)	Log Treatment	Treatment (ng/mg mosquito)	Alive	Total	% Mortality - Abbott Corrected
0.000		0.000	9.667	10.000	**0.000**
0.040	-1.740	0.018	9.000	10.000	**6.897**
0.400	-0.740	0.182	9.667	10.000	**0.000**
4.000	0.260	1.818	4.667	10.000	**51.724**
10.000	0.658	4.545	2.667	10.000	**72.414**
20.000	0.959	9.091	1.000	10.000	**89.655**
40.000	1.260	18.182	0.333	10.000	**96.552**

Topical Treatment: Etofenprox

Strain: ORL

Treatment Active Ingredient (ng)	Log Treatment	Treatment (ng/mg mosquito)	Alive	Total	% Mortality - Abbott Corrected
0.000		0.000	9.167	10.000	**0.000**
0.004	-2.653	0.002	9.000	10.000	**1.818**
0.020	-1.954	0.011	8.333	10.000	**9.091**
0.040	-1.653	0.022	6.667	10.000	**27.273**
0.200	-0.954	0.111	0.333	10.000	**96.364**
0.400	-0.653	0.222	0.000	10.000	**100.000**
4.000	0.347	2.222	0.000	10.000	**100.000**

Topical Treatment: Etofenprox

Strain: PR F7

Treatment Active Ingredient (ng)	Log Treatment	Treatment (ng/mg mosquito)	Alive	Total	% Mortality - Abbott Corrected
0.000		0.000	9.667	10.000	**0.000**
0.040	-1.740	0.018	9.667	10.000	**0.000**
0.400	-0.740	0.182	9.333	10.000	**3.448**
4.000	0.260	1.818	8.000	10.000	**17.241**
10.000	0.658	4.545	7.000	10.000	**27.586**
20.000	0.959	9.091	4.000	10.000	**58.621**
40.000	1.260	18.182	1.667	10.000	**82.759**
200.000	1.959	90.909	0.000	10.000	**100.000**

Topical Treatment: Deltamethrin
Strain: ORL

Treatment Active Ingredient (ng)	Log Treatment	Treatment (ng/mg mosquito)	Alive	Total	% Mortality - Abbott Corrected
0.00000		0.000	9.667	10.000	**0.000**
0.00004	-4.653	0.000	9.000	10.000	**6.897**
0.00020	-3.954	0.000	7.333	10.000	**24.138**
0.00040	-3.653	0.000	3.000	10.000	**68.966**
0.00200	-2.954	0.001	2.667	10.000	**72.414**
0.02000	-1.954	0.011	0.000	10.000	**100.000**
0.20000	-0.954	0.111	0.000	10.000	**100.000**

Topical Treatment: Deltamethrin
Strain: PR F9

Treatment Active Ingredient (ng)	Log Treatment	Treatment (ng/mg mosquito)	Alive	Total	% Mortality - Abbott Corrected
0.000		0.000	8.500	10.000	**0.000**
0.004	-2.740	0.002	7.333	9.000	**13.725**
0.040	-1.740	0.018	7.333	10.000	**13.725**
0.400	-0.740	0.182	3.667	10.000	**56.863**
4.000	0.260	1.818	0.000	10.000	**100.000**
10.000	0.658	4.545	0.000	10.000	**100.000**
20.000	0.959	9.091	0.000	10.000	**100.000**
40.000	1.260	18.182	0.000	10.000	**100.000**

Topical Treatment: DDT
Strain: ORL

Treatment Active Ingredient (ng)	Log Treatment	Treatment (ng/mg mosquito)	Alive	Total	% Mortality - Abbott Corrected
0		0.000	9.667	10.000	**0.000**
0.156	-1.107	0.078	8.333	10.000	**13.793**
0.313	-0.806	0.156	7.333	10.000	**24.138**
0.625	-0.505	0.313	5.667	10.000	**41.379**
1.250	-0.204	0.625	5.333	10.000	**44.828**
2.500	0.097	1.250	0.333	10.000	**96.552**
5.000	0.398	2.500	0.000	10.000	**100.000**

Topical Treatment: DDT
Strain: PR F9

Treatment Active Ingredient (ng)	Log Treatment	Treatment (ng/mg mosquito)	Alive	Total	% Mortality - Abbott Corrected
0.000		0.000	8.500	10.000	**0.000**
0.2	-0.954	0.111	7.333	10.000	**13.725**
2	0.046	1.111	5.667	10.000	**33.333**
20	1.046	11.111	5.667	10.000	**33.333**
50	1.444	27.778	1.333	10.000	**84.314**
100	1.745	55.556	1.000	10.000	**88.235**
200	2.046	111.111	0.000	10.000	**100.000**

Topical Treatment: Transfluthrin

Strain: ORL

Treatment Active Ingredient (ng)	Log Treatment	Treatment (ng/mg mosquito)	Alive	Total	% Mortality - Abbott Corrected
0.000		0.000	9.667	10.000	**0.000**
0.004	-2.653	0.002	10.000	10.000	**-3.448**
0.020	-1.954	0.011	9.333	10.000	**3.448**
0.040	-1.653	0.022	8.333	10.000	**13.793**
0.200	-0.954	0.111	4.667	10.000	**51.724**
0.400	-0.653	0.222	1.667	10.000	**82.759**
4.000	0.347	2.222	0.000	10.000	**100.000**

Topical Treatment: Transfluthrin

Strain: PR F9

Treatment Active Ingredient (ng)	Log Treatment	Treatment (ng/mg mosquito)	Alive	Total	% Mortality - Abbott Corrected
0.000		0.000	8.500	10.000	**0.000**
0.040	-1.699	0.020	8.333	10.000	**1.961**
0.400	-0.699	0.200	7.000	10.000	**17.647**
4.000	0.301	2.000	5.667	10.000	**33.333**
10.000	0.699	5.000	2.667	10.000	**68.627**
20.000	1.000	10.000	2.333	10.000	**72.549**
40.000	1.301	20.000	0.333	10.000	**96.078**
