# Supplementary material for: Pyrethroid resistance alters the blood-feeding behavior in Puerto Rican Aedes aegypti mosquitoes exposed to treated fabric
Source: PLoS Negl Trop Dis. 2017 Sep 20;11(9):e0005954. doi: 10.1371/journal.pntd.0005954 (PMC5624645; doi:10.1371/journal.pntd.0005954)
Supplement: S2 Table — (PDF) [file pntd.0005954.s002.pdf]

S2 Table. Percent bite protection by treatment type and mosquito strain for all replicates combined in the blood-feeding bioassays.

Sleeve Treatment: Permethrin
Strain: ORL

Treatment (mg/cm²)	Bloodfed	Total	Prop.	% Bite Protection
0	50.33	58.00	0.87	0.00
0.000072	35.17	60.17	0.58	32.65
0.00072	26.00	57.33	0.45	47.74
0.011	13.00	54.67	0.24	72.60
0.22	6.67	56.83	0.12	86.48
0.97	2.33	54.67	0.04	95.08
2.60	0.80	58.60	0.01	98.43

Sleeve Treatment: Permethrin
Strain: PR F5-7

Treatment (mg/cm²)	Bloodfed	Total	Prop.	% Bite Protection
0	41.83	51.83	0.81	0.00
0.000072	29.00	45.33	0.64	20.74
0.00072	28.33	44.67	0.63	21.40
0.011	23.17	47.00	0.49	38.93
0.22	20.17	45.67	0.44	45.28
0.97	10.67	47.50	0.22	72.18
2.60	4.00	44.20	0.09	88.79

Sleeve Treatment: Etofenprox
Strain: ORL

Treatment (mg/cm²)	Bloodfed	Total	Prop.	% Bite Protection
0	51.33	58.00	0.89	0.00
0.000072	31.67	58.67	0.54	39.01
0.00072	27.33	58.83	0.46	47.51
0.099	13.50	57.50	0.23	73.47
0.30	11.17	61.33	0.18	79.43
1.17	5.67	60.67	0.09	89.45
18.6	5.75	57.25	0.10	88.65

Sleeve Treatment: Etofenprox
Strain: PR F5-7

Treatment (mg/cm²)	Bloodfed	Total	Prop.	% Bite Protection
0	40.00	50.67	0.79	0.00
0.00072	34.83	49.83	0.70	11.46
0.0036	25.50	50.17	0.51	35.61
0.099	25.00	51.83	0.48	38.91
0.30	15.00	49.67	0.30	61.74
1.17	16.83	47.33	0.36	54.95
1.82	21.67	61.00	0.36	55.01

Sleeve Treatment: Deltamethrin
Strain: ORL

Treatment (mg/cm²)	Bloodfed	Total	Prop.	% Bite Protection
0	97.75	109.50	0.89	0.00
0.000072	54.83	112.83	0.49	45.56
0.00072	43.67	113.50	0.38	56.90
0.011	14.17	108.50	0.13	85.37
0.22	11.50	101.50	0.11	87.31
0.48	10.50	114.00	0.09	89.68
2.60	7.80	92.00	0.08	90.50

Sleeve Treatment: Deltamethrin
Strain: PR F6-8

Treatment (mg/cm²)	Bloodfed	Total	Prop.	% Bite Protection
0	57.00	59.00	0.97	0.00
0.000072	51.00	59.00	0.86	10.53
0.00072	45.00	58.00	0.78	19.69
0.011	38.00	65.00	0.58	39.49
0.22	31.00	64.00	0.48	49.86
0.48	21.00	62.00	0.34	64.94
2.60	17.00	56.00	0.30	68.58

Sleeve Treatment: DDT
Strain: ORL

Treatment (mg/cm^2)	Bloodfed	Total	Prop.	% Bite Protection
0	97.75	109.50	0.89	0.00
0.000072	57.17	105.83	0.54	39.49
0.00072	74.50	115.33	0.65	27.64
0.011	55.33	110.00	0.50	43.65
0.22	46.00	102.83	0.45	49.89
0.97	44.50	109.67	0.41	54.54
2.60	24.80	94.20	0.26	70.51

Sleeve Treatment: DDT
Strain: PR F6-8

Treatment (mg/cm^2)	Bloodfed	Total	Prop.	% Bite Protection
0	51.17	58.67	0.87	0.00
0.000072	46.83	60.50	0.77	11.24
0.00072	38.50	58.00	0.66	23.89
0.011	35.17	56.67	0.62	28.84
0.22	35.33	63.83	0.55	36.53
0.97	31.50	55.67	0.57	35.12
2.60	21.00	59.60	0.35	59.60
